# Pre-clinical Research on Bladder Toxicity After Radiotherapy for Pelvic Cancers: State-of-the Art and Challenges

**DOI:** 10.3389/fonc.2020.527121

**Published:** 2020-10-22

**Authors:** Stefania Zuppone, Andrea Bresolin, Antonello E. Spinelli, Giuseppe Fallara, Roberta Lucianò, Federico Scarfò, Fabio Benigni, Nadia Di Muzio, Claudio Fiorino, Alberto Briganti, Andrea Salonia, Francesco Montorsi, Riccardo Vago, Cesare Cozzarini

**Affiliations:** ^1^Division of Experimental Oncology, Urological Research Institute, IRCCS San Raffaele Scientific Institute, Milan, Italy; ^2^Fondazione Centro San Raffaele, Milan, Italy; ^3^Department of Medical Physics, IRCCS San Raffaele Scientific Institute, Milan, Italy; ^4^Experimental Imaging Center, IRCCS San Raffaele Scientific Institute, Milan, Italy; ^5^Unit of Pathology, IRCCS San Raffaele Scientific Institute, Milan, Italy; ^6^Department of Radiotherapy, IRCCS San Raffaele Scientific Institute, Milan, Italy; ^7^University Vita-Salute San Raffaele, Milan, Italy

**Keywords:** small animal, pre-clinical research, urinary bladder toxicity, micro-irradiator, animal model

## Abstract

Despite the dramatic advancements in pelvic radiotherapy, urinary toxicity remains a significant side-effect. The assessment of clinico-dosimetric predictors of radiation cystitis (RC) based on clinical data has improved substantially over the last decade; however, a thorough understanding of the physiopathogenetic mechanisms underlying the onset of RC, with its variegated acute and late urinary symptoms, is still largely lacking, and data from pre-clinical research is still limited. The aim of this review is to provide an overview of the main open issues and, ideally, to help investigators in orienting future research. First, anatomy and physiology of bladder, as well as the current knowledge of dose and dose-volume effects in humans, are briefly summarized. Subsequently, pre-clinical radiobiology aspects of RC are discussed. The findings suggest that pre-clinical research on RC in animal models is a lively field of research with growing interest in the development of new radioprotective agents. The availability of new high precision micro-irradiators and the rapid advances in small animal imaging might lead to big improvement into this field. In particular, studies focusing on the definition of dose and fractionation are warranted, especially considering the growing interest in hypo-fractionation and ablative therapies for prostate cancer treatment. Moreover, improvement in radiotherapy plans optimization by selectively reducing radiation dose to more radiosensitive substructures close to the bladder would be of paramount importance. Finally, thanks to new pre-clinical imaging platforms, reliable and reproducible methods to assess the severity of RC in animal models are expected to be developed.

## Introduction

Despite dramatic advance in pelvic radiotherapy, mainly due to the implementation of image-guided intensity-modulated (IMRT) techniques, acute and late urinary toxicity (radiation cystitis [RC] or actinic cystitis) remains a significant side-effect, especially in the case of high-dose schedules such as those used for prostate and gynecological cancer treatment (1, 2). The assessment of clinical, molecular and/or genetic predictors of urinary toxicity has improved substantially over the last decade, also by use of data from large cohorts of prospectively monitored patients treated with external beams or brachytherapy (3–7). Nevertheless, a thorough understanding of the pathophysiology at the base of acute and late radiation-induced urinary symptoms, such as urgency, nocturia, urethral stenosis, incontinence, hematuria, etc., is still largely lacking, as well as robust pre-clinical data based on animal models. The advent of micro-irradiators, capable of delivering radiotherapy even to small animals with micrometric resolution, and the simultaneous rapid advancement of imaging methods, might lead to big advancements into this field. Animal models of radiation-induced bladder toxicity might improve the current understanding of physio-pathogenetic mechanisms at the base of radiation induced cystitis and expedite the detection and testing of possible radioprotective agents aimed at reducing such damage.

The aim of the current paper is therefore to review this suboptimally explored field of research, with the aim of providing both basic researchers and radiation oncologists an overview of the main open issues and, ideally, to assist them in orienting future research. First, normal bladder anatomy and physiology, radiation dose and dose-volume effects are briefly summarized. Then, the potential of modern radiobiology “tools” and the realization of robust and reproducible animal models of radiation-induced cystitis, are described. The most promising approaches aimed at preventing/minimizing RC are then discussed, by systemically reviewing both historical and recent findings on animal experiments. Finally, suggestions for future research will be explored.

### Anatomical Features

The urinary bladder collects urine from the ureters and, when sufficiently filled, empties through the urethra. Two different parts can be distinguished: the bladder body, located above the inter-ureteric crest, and the base, composed of the trigone, the bladder neck and the urethro-vesical junction (8).

The urinary bladder is a hollow smooth muscle organ made up of 4-folds. The most external one is the adventitia, a serous layer. Below, the detrusor muscle, a thick muscular layer made up of smooth muscle cells and extracellular matrix, rich in collagen and elastin, allows bladder emptying. Three layers of muscular cells, differently distributed between bladder body and neck, exists: outer longitudinal, circular medial and inner longitudinal (8, 9). The submucosa is the smooth connective tissue laying between the detrusor muscle and the inner mucosal layer. It is rich in elastin and collagen, mostly types 1 and 3, mixed with a proteoglycan matrix which attracts water, giving the tissue high elasticity (8). Finally, the most internal layer, the mucosa is structured in three parts from the outer to the inner: muscolaris mucosae, a thin muscular layer dividing the submucosa from the mucosa; lamina propria, a connective layer, rich in blood vessels and nerve endings, which structurally and functionally supports the urothelium; and urothelium, a pseudostratified epithelium where basal, intermediate and umbrella cells can be identified (8). Each umbrella cell covers many intermediate cells, and their shape resembles an umbrella; they are in direct contact with urine and flatten when the bladder fills (10). Most of their membrane apical surface (almost 80%) is covered with protein plaque whose precise composition is unclear, but a main component seems to be protein called Uroplakin (9). Together with the glycosaminoglycan (GAG) layer over the urothelium and the tight junctions between umbrella cells, the protein plaque creates the urine-plasma barrier and probably hampers bacterial adherence (9, 11). Another function of urothelial cells seems the detection of bladder volumes and strain, through a direct signaling on afferent nerves or indirect communication with interstitial cells (9).

### Physiology and Mechanical Features

Storage of urine and voiding represent the two most important functions of the urinary bladder, involving extremely complex interactions between its structural components and the nervous system.

Urine storage occurs at low pressure, and the bladder behaves passively (8, 9). During filling, the smooth muscle cells have to relax, elongate and rearrange. Laplace's law, assuming spherical shape, incompressible wall and an isotropic homogeneous stretch, accurately describes the bladder mechanics during filling: wall tension, intravesical pressure and bladder size are directly related (8, 9). During bladder filling, intravesical pressure is relatively constant, avoiding urine outflow to the upper urinary tract, and bladder is slowly stretched while volume increases (8, 9, 11, 12). A small increase in bladder pressure during filling is caused by a small increase in bladder wall tension, due to the viscoelastic response of the extracellular matrix when collagen fibers, initially folded, begin to stretch (12, 13). The viscoelastic property of the bladder wall is directly reflected in bladder compliance (*C*), defined as the change in volume (*V*) relative to the corresponding change in intravesical pressure (*P*). High compliance indicates that bladder volume could increase during filling without a significant pressure surge (9).

During the active micturition phase, smooth cells contract rapidly and synchronously throughout the bladder (8, 9). Immediately prior to voiding, after parasympathetic nerve system activation, the sphincters relax, the detrusor contracts and internal pressure increases (9, 12, 14). Contraction of muscle cells occurs with the interaction of α-myosin and actin molecules, triggered by intracellular calcium concentration increase and calmodulin activation. Thanks activation of muscarinic M3 receptor by acetylcholine, intracellular calcium is released by the opening of membrane nifedipine-sensitive L-type Ca2+ channels, by the increase in inositol 1,4,5-trisphosphate (IP3) production with consequent release of calcium from the sarcoplasmic reticulum, and by the activation of ryanodine receptors (9).

In addition, cellular framework and membrane attachments are provided by other cytoskeletal proteins, such as non-muscle β- and γ-actins, filamin, calponin and intermediate filaments (8, 9, 11).

All of these mechanisms can be significantly altered and impaired by irradiation (See below 2.5.2 Radiation damage and bladder dysfunction).

### Clinical Doses and Thresholds in Humans

Although both state-of-the-art imaging guidance and intensity modulated techniques have been developed to allow better radiation dose distribution and improve treatment safety, when radiation is delivered to pelvic organs, the involvement of healthy portions of the bladder is inevitable. Therefore, a significant fraction of irradiated patients experience bladder radiation-induced side effects. The onset of RC significantly affects patients' quality of life, as there are no recommended standard management treatments (15). Radiation dose, fraction, and field size, as well as age at radiation treatment, genetic variations, concurrent therapies and comorbidities such as diabetes and immunodeficiency are considered risk factors for developing RC (16).

In particular, several recent reviews (17–20) have outlined how radiation dose correlates to the risk of urinary toxicity. Evidence of a quite rapid increase of the risk of Grade 3 urinary toxicity according to the Common Toxicity Criteria for Adverse Events (CTCAE, e.g., urethral stenosis and/or bladder neck stricture requiring surgical intervention, gross hematuria requiring blood transfusion and/or hyperbaric oxygen therapy, urinary incontinence requiring treatments such as invasive treatment) (21) for 2-Gy equivalent doses (EQD2) to the whole bladder above 50–55 Gy have been demonstrated (22). Segments of the urinary tract can receive much higher doses of radiation during bladder, prostate and gynecologic cancer radiotherapy, and dose-volume effects for several urinary symptoms have been demonstrated (23). The bladder shows the behavior of a prevalently serial organ, being extremely sensitive to even small volumes receiving high doses, such that any procedure leading to a reduction of bladder volumes receiving EQD2 doses ≥75–78 Gy or ≥8–12 Gy/week may significantly decrease the risk of toxicity (18). Here, image-guided radiotherapy (IMRT) reduced bladder areas overlaying the planning target volume (PTV) and hence lowered urinary toxicity risk. A spatial effect was also highlighted in the trigone, the most radiosensitive bladder substructure, for which the mean dose delivered was proven to be strongly associated to a higher risk of severe acute and late urinary damage (19). More recently, growing evidence of bladder sensitivity to fractionation suggested an $\frac{\alpha }{\beta }$ value (a parameter of the sensitivity of both tumor and healthy tissues to fractionation) significantly lower than previously hypothesized, in the range of 1 Gy. The prevalent dose-effect in hypofractionated protocols is consistently associated with the risk of severe late toxicities such as gross hematuria, urethral stenosis and severe incontinence (21), a risk which rises considerably for prescribed EQD2 radiation doses to the PTV above 80–85 Gy (calculated for an $\frac{\alpha }{\beta }$ ratio of 1 Gy).

### Image-Guided Small Animal Irradiation Systems

The use of image-guided small animal irradiation systems is rapidly increasing in preclinical and translational radiotherapy research (24, 25). Recent technological developments allow the possibility of mimicking *in vivo* the main steps of clinical image-guided radiotherapy, from CT images to treatments, and the evaluation of the effects of radiation on tumor and healthy tissues. The main difference with respect to the clinical setting is that the entire procedure, comprising CT imaging, dose planning and delivery, is performed within about 20 min, while the animal is under anesthesia. This strict time limitation is necessary to reduce the effect of hypothermia, as well as to increase the number of animals that can be treated in a single experimental session.

Considering the size of the animals and the small volumes to be treated, lower photon energy beams generated using a conventional x-ray tube working at a tension up to about 200–250 kVp (instead of MV energies needed for treating humans) are used. The same x-ray tube is normally employed to acquire CT images of the animal, with a tension range between 40 and 80 kVp.

Two small animal image guided irradiators are currently commercially available: SARRP (Xstrahl, Atlanta, GA, USA) and XRAD225Cx SmART (PXI North Branford, CT, USA). The two systems are similar in terms of x-ray energy and differ mainly in terms of the geometry of CT acquisition. There are also home-made solutions and prototypes developed by several research groups (26–29). An exhaustive description of these prototypes is beyond the scope of this review.

Given the size of mice and rats, the downscaling of the imaging, planning and dose delivery procedures on such small animals is not a trivial issue. As mentioned in a recent ESTRO ACROP guideline (30), challenges include how to perform accurate and precise small field dosimetry and how to verify dose distributions on such small fields.

**Figure 1:**
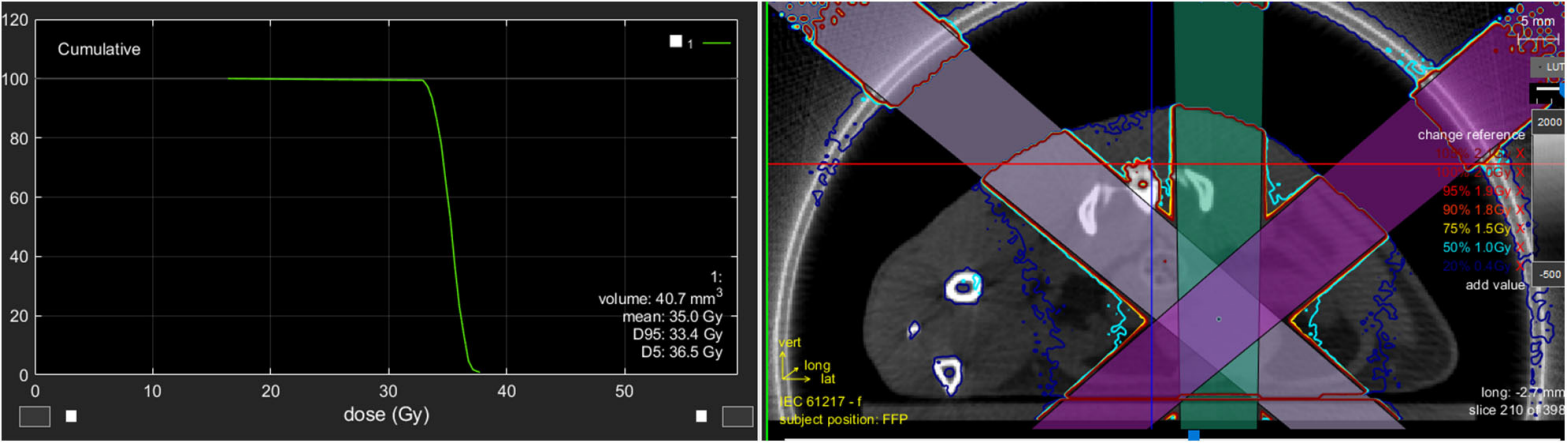
The image shows an example of a planned treatment to the entire rat bladder. The TPS allows the calculation of dose volume histogram (DVH), the visualization of the beams, the dose distributions, etc., similar to a clinical TPS.

With regard to the two available commercial systems, the dose is calculated using dedicated treatment planning systems (TPS) based on Superposition–Convolution (31) and Monte Carlo simulation (25). An example of a planned treatment to the entire rat bladder is shown in Figure 1. Here, the TPS allows the calculation of dose volume histogram (DVH), visualization of the beams, dose distributions etc., as in clinical TPS.

In order to obtain a better delineation of the target volume it is also possible to merge the planning CT image with images acquired with different modalities, such as Magnetic Resonance (MR) (32), Positron emission tomography (PET) (33), single photon emission tomography (SPECT) (34) and bioluminescence imaging (35, 36). Importantly, the use of multimodal imaging can significantly increase planning time, while reducing system throughput.

## Literature Review

### Methods of Bibliographic Research

In October 2019, the peer-reviewed scientific literature was scrutinized by S.Z. and A.B. for pre-clinical research on *in-vivo* small animal (mouse and rat) models of radiation cystitis. The research platform *Scopus* (Elsevier tool) was used: the search strategy and the multiple keywords combinations used are detailed in Table 1. Eligibility was limited to documents in the medical area published in English. Specific exclusion criteria were used to avoid non-pertinent subjects, such as studies relative to bladder cancer, radioactive nuclides or non-ionizing radiation, *in vitro* experiments, pharmaceutical or clinical trials. Of the initial 78 abstracts reviewed, 30 were excluded on the base of the above mentioned criteria, resulting in 48 full papers (4 of which are reviews) published between 1985 and 2019. The articles, despite reporting very different end-points and methods, are grouped into three main topics (i.e., Radiation damage and bladder dysfunction; Pathology and preclinical models; Radioprotective agents) and are summarized in Tables 2–4, respectively. Table 5 includes five studies on abscopal/bystander effects retained for completeness, although they are not discussed in the current review.

**Table 1:** Search and exclusion strategy used in the bibliographic research on scopus for the current review.

**Search and exclusion strategy**	**Input in the research platform Scopus**
**Search step 1**	
Multiple search in titles, abstracts and keywords of the following subjects:	TITLE-ABS-KEY(
3. Pre-clinical small animal research	“preclinical” OR “rat” OR “mice”
4. External radiotherapy (X-rays therapeutic beam)	“radiotherapy” OR “radiation injuries” OR “radiation dose” OR “radiation-protective agents” OR “ionizing radiation”
5. Urinary tract	“bladder” OR “urethra”
6. Models of radio-induced toxicity	“model” OR “tolerance” OR “toxicity” OR “controlled study” OR “dose response”)
**Exclusion step 1**	
Restriction to the only medical subject area	SUBJAREA (medi)
**Exclusion step 2**	
Limitation to works published in english	LIMIT-TO (LANGUAGE, “English”)
**Exclusion step 3**	
Reinforcement of the exclusion criteria for subjects outside the scope of the current review:	AND NOT(
7. Bladder cancer	“bladder cancer” OR “bladder carcinoma”
8. Clinical studies	OR “clinical trials” OR “case report”
9. *In vitro* experiments	OR “*in vitro*”
10. Internal radiotherapy	OR “radionuclide” OR “radioactivity” OR “PET” OR “intraoperative”
11. Pharmaceutical studies	OR “radiopharmaceutical” OR “pharmacodynamics”
12. Non-ionizing radiation	OR “electromagnetic”)

*At the end of the first examination through the evaluation of 78 abstracts, 30 papers were excluded based on the established criteria, resulting in 48 full papers (4 of which are reviews) published in the period 1985–2019*.

**Table 2:** Chronological summary of the pre-clinical cystometric studies about radio-induced toxicity on the normal bladder.

**References**	**Animal model (strain)**	**Dose set-up**	**Endpoint (method)**	**Toxicity timing after RT**	**Findings**
Knowles et al. (37)	Female rat (Wistar)	20-40 Gy in 1 fr. to ureter/trigone delivered by 300 kV X-rays machine through a ventral beam	Hydronephrosis (intravenous urography)	Death: <40 days Hydronephrosis: >42 days	Rate at 23.4 Gy to ureter = 14/16 Rate at 25 Gy to trigone = 9/11 Many rats died with 37.4 Gy to ureter; No death associated with 40 Gy to trigone
Lundbeck et al. (38)	Female Mouse (C_3_D_2_F_1_/Bom)	20 Gy in 1 fr. delivered by 250 kV X-rays machine	Reservoir function (transurethral cystometry)	No change in the control group within 200 days. Biphasic change in the irradiated group	Evidence of biphasic change in the bladder reservoir function: acute and late damage
Lundbeck et al. (39)	Female mouse (C_3_D_2_F_1_/Bom)	5–30 Gy in 1 fr. delivered by 250 kV X-ray machine through a ventral beam	Reservoir function (transurethral cystometry)	Acute response: 10–14 days (Functions restored after another month) Late response: dependent on the dose	RD_50_ = 17.2 Gy for the acute response. Late toxicity time was dose-dependent: 10–15 Gy, 20 Gy, 25–30 Gy groups were significant different
Stewart et al. (40)	Female mouse (C3H/Hen Af-nu^+^)	8–16 Gy repeated after 1 day or 3 or 9 months and delivered by 250 kV X-ray machine through a ventral beam	Functional damage (transurethral cystometry)	Early damage: 2 weeks (reirradiation at 9 months after 16Gy) Late damage: undirect relationship with the dose administered in the first treatment and no dependency upon time between treatments	Prolonging the overall treatment time does not result in the prevention of late radiation injury in the bladder
Stewart et al. (41)	Female mouse (C3H/Hen Af-nu^+^)	10–30 Gy in 1 fr. delivered by 250 kV X-ray machine through a ventral beam	Functional damage (transurethral cystometry)	Acute response: 5–21 days (duration: <1 week) Late response: 16–40 weeks	Acute response rate (20–30 Gy): 20–40% Late response rate (10–15 Gy): <20%
Bentzen et al. (42)	Female mouse (C_3_D_2_F_1_/Bom)	1 to 10 fractions for an overall time of 4–4.5 days and a total dose of 5-60 Gy delivered by 250 kV X-ray machine	Reservoir function (transurethral cystometry)	Late response: >30 days Latent period: 35–401 days	α/β = 5.8 Gy Late radiation injury in the mouse urinary bladder was not highly sensitive to change in dose per fraction
Dörr et al. (43)	Female Mouse (C3H/Neu)	Single-dose or fractionated irradiation delivered by Seifert Isovolt 320/20 X-ray machine	Reservoir function (transurethral cystometry)	Early response 7–25 days after ≥10 Gy in 1 fr. Duration of the response: 3–9 days	α/β = = 11.1-12.4 Gy (acute responding tissue)
Vale et al. (44)	Female rat (Wistar)	10, 15, 20, 25 Gy in 1fr. delivered by Pantak 320- kV X-ray generator	Reservoir function (transurethral cystometry)	First reduction: 4 weeks Second reduction: 3–4 months and persistent at 6 months	Biphasic reduction for 15–25 Gy
Dörr et al. (45)	Female Mouse (C3H/Neu)	Four equal-sized dose fractions were applied with intervals of 0–8 h and delivered by Seifert Isovolt 320/20 X-ray machine	Reservoir function (transurethral cystometry)	Acute response: <30 days Half-time of repair = 1.2 h	ED50 = 18.2 Gy for single dose ED50 = 28.1 Gy for 8h protocol $\frac{\alpha }{\beta }$ = 10.4 Gy
Dörr et al. (46)	Female mouse (C3H/Neu)	19 Gy in 1 fr. delivered by Seifert Isovolt 320/20 X-ray machine	Reservoir function (transurethral cystometry)	Complete recovery <30 days, followed by a symptom-free latent time of about 15 weeks	No changes in the diurnal pattern were observed. In the late phase, the absolute capacity and the amplitude of fluctuations decreased
Dörr et al. (47)	Female mouse (C3H/Neu)	Graded single dose delivered by Seifert Isovolt 320/20 X-ray machine	Reservoir function (transurethral cystometry)	Acute response:- 1–15 days (I wave) with mean latent time = 7.1 days- 16–30 days (II wave) with mean latent time = 23.3 days	ED50 = 21.7 Gy (I acute wave) ED50 = 19.3 Gy (II acute wave) ED50 = 18.7 Gy (late response) Response during the second but not the first acute wave correlated with the late response (*p* = 0.0008)
Dörr et al. (48)	Female Mouse (C3H/Neu)	(i) 1 to 10 fr. applied within 5 days.(ii) 4 equal-sized dose fractions applied with intervals of 0–8 h and delivered by Seifert Isovolt 320/20 X-ray machine	Reservoir function (transurethral cystometry)	Half time of repair: 0.39 h Latent time to chronic functional changes: 12–40 weeks inversely dependent on the BED	(i) repair capacity: $\frac{\alpha }{\beta }$ = 4.4 Gy (ii) repair kinetics: $\frac{\alpha }{\beta }=$ 3.7 Gy Dose fractionation sparing effect was in the lower range of tissues with a chronic response
Jaal et al. (49, 50)	Female Mouse (C3H/Neu)	20 Gy in 1fr. delivered by Seifert Isovolt 320/20 X-ray machine through a ventral beam	Reservoir function (transurethral cystometry)	Rate = 40% for days 0–15 Rate = 64% for days 16–30 Rate = 71% after 180 days	Irradiation induced significant acute and chronic reduction in bladder capacity by >50%
Rajaganapathy et al. (51)	Female rat (Sprague-Dawley)	20, 30, 40 Gy in 1fr. delivered by SARRP unit through three ventral beams	Micturition frequency (metabolic cage)	Early response: 6 weeks	40 Gy caused reductions in the mean inter-micturition interval by ~20 min
Zwaans et al. (52)	Female Mouse (C3H/HeN)	20 Gy in 1fr. delivered by SARRP unit through two ventral beams	Micturition frequency (metabolic cage)	Late response: starting at 17 weeks	Micturition frequency in irradiated mice was significantly increased compared to controls. The radiation exposure attenuated the urothelial integrity long-term
Giglio et al. (53)	Female rat (Sprague–Dawley)	20 Gy in 1fr. delivered by 6 MeV linac through two side- field	Functional damage (metabolic cage)	14 days	Irradiation led to urodynamic changes. Water intake and micturition frequency were found not to be correlated

*In these studies the endpoint is the functional damage in terms of reservoir function (reduction in the bladder capacity by >50% at a fixed intravesical pressure) and/or micturition frequency*.

*RT, radiotherapy; RD_50_, response dose 50%; ED50, radiation dose producing damage in the 50% of cases; dose BED, biologically effective dose; H&E, Hematoxylin & Eosin; SARRP, small animal radiation research platform*.

**Table 3:** Chronological summary of the pre-clinical immunohistochemical studies about radio-induced toxicity on the normal bladder.

**References**	**Animal model (strain)**	**Dose set-up**	**Endpoint (method)**	**Toxicity timing after RT**	**Findings**
Stewart et al. (41)	Female mouse (C3H/Hen Af-nu^+^)	10-30 Gy in 1 fr. delivered by a 250 kV X-rays machine through a ventral beam	Morphological changes (hematoxylin eosin staining)	2 weeks: no changes 7–12 months: epithelial denudation, hyperplasia, necrosis, fibrosis	The late damage was characterized by epithelial denudation and focal hyperplasia; fibrosis and ulceration were also detectable at higher doses (20–30 Gy)
Vale et al. (44)	Female rat (Wistar)	10, 15, 20, 25 Gy in 1fr. delivered by Pantak 320- kV X-ray generator	Morphological changes (H&E, toluidine blue staining)	6 months	Evidence of increase mast cell density. Fibrosis in 9/18 rats
Crowe et al. (54)	Female rat (Wistar)	15 and 25 Gy in 1 fr. delivered by Pantak HF 320 X-ray generator	Changes in neuropeptides	6 months	Increase in the density of NPY, SP- and TH-immunoreactive nerves in the urinary bladder
Kraft et al. (55)	Mouse (sex n.a.) (C3H/Hen Af-nu+ and C3H/Neu)	25 or 19 Gy (ED80 40 weeks after RT)	Morphological changes (TGF-β expression and collagen content)	Increase in TGF-β: 90–360 days Increase in collagen I and III: >180 days	TGF-beta expression and connective tissue metabolism were important factors determining reduced bladder function after irradiation
Kruse et al. (56)	Female mouse (C3H/Hen Af-nu^+^)	20 Gy to rectum 16 Gy to kidney delivered in 1 fr. by 250-kV X-ray	Telangiectasia (microarray analysis of RNA isolated from pre-irradiated kidney/ rectum)	10–20 weeks	Identification of genes expressed in tissues with manifest vascular damage
Kanai et al. (57)	Rat (Sprague-Dawley) Mouse (nNOS^−/−^, iNOS^−/−^, eNOS^−/−^, C57BL10)	0–50 Gy in 1 or more fr. (1–3 days interval) delivered by 6 MeV linac	Umbrella cells ulceration	n.a.	mtNOS was in the cardiomyocytes and urothelial cells, and can be either protective or detrimental
Jaal et al. (49, 50)	Female mouse (C3H/Neu)	20 Gy in 1fr. delivered by Seifert Isovolt 320/20 X-ray machine	Morphological changes (ICAM-1 expression)	Increasing signal at day 2–4 and 16–28 Permanent signal between 90–360 days	Irradiation induces significant early and late deregulation in ICAM-1 expression levels, preceding bladder functional response
Jaal et al. (58)	Female Mouse (C3H/Neu)	20 Gy in 1fr. delivered by Seifert Isovolt 320/20 X-ray machine	Vasodilatation (COX-2 in blood vessels)	Early: 4–16 days Late: 90–360 days	COX-2 dependent inflammatory response in the bladder wall during the early phase after radiation
Jaal et al. (59)	Female mouse (C3H/Neu)	20 Gy in 1fr. delivered by Seifert Isovolt 320/20 X-ray machine	Decrease in n° of umbrella cells (UP-III)	Early phase: 0–31 days Initial late phase: 90, 120 days	Irradiation resulted in morphological impairment of the urothelial barrier
Jaal et al. (60)	Female Mouse (C3H/Neu)	20 Gy in 1fr. delivered by Seifert Isovolt 320/20 X-ray machine	Amount of collagen (Masson's Trichrome)	In the entire late phase, but most pronounced at day 120 and 180	Suggested neovascularization in the late phase of radiation-induced bladder damage
Soler et al. (61)	Female rat (Lewis)	20 Gy in 1fr. delivered by Cesium isotope-based irradiator collimated by shield on bladder	Amount of collagen (Masson's Trichrome) and vascularization (VonWillebrand factor)	1.5 and 3 months	Anti-Angiogenesis therapy is proposed to prevent and/or treat the pathology of radiation cystitis
Xu et al. (62)	Male mouse (NCRNU)	5 Gy in 5 fr. delivered by 250 kV X-ray machine	Ultrastructural and mitochondrial damage	60 days	Parthenolide enhanced radiosensitivity of prostate tumors but protects healthy tissues (bladder) from radiation
Ozbilgin et al. (63)	Male mouse (Swiss Albino)	10 Gy in 1 fr. delivered by Co^60^ RT	Morphological changes (H&E), POMC immunoreactivity	24 h, 48 h, and 7 days	No morphological alterations. Expression of POMC on the urothelium seems to spare bladder from radiation injuries
Ozbilgin et al. (64)	Male mouse (Swiss Albino)	10 Gy in 1 fr. delivered by Co^60^ RT	Reaction of versican and HB-EGF	7 days	Increase of versican and HB-EGF concentrations may play a role in the side effects of RT
Ozbilgin et al. (65)	Male mouse (Swiss Albino)	10 Gy in 1 fr. delivered by Co^60^ RT	COX-1 and COX-2 immunoreactivity	24 h, 48 h, and 7 days	The expression of COX-1 and COX-2 seems to prevent bladder damage from radiation
Giglio et al. (53)	Female rat (Sprague–Dawley)	20 Gy in 1fr. delivered by 6 MeV linac through two side- field	Extensive immuno-histochemical characterization	16 h−14 days	Irradiation may suppress important immunoregulatory pathways
Rajaganapathy et al. (51)	Female rat (Sprague-Dawley)	20, 30, 40 Gy in 1fr. delivered by SARRP unit through three ventral beams	Morphological changes (H&E)	Early response: 6 weeks	Evidence of degenerative type epithelial changes, urothelial swelling and hyperplasia
Zwaans et al. (52)	Female Mouse (C3H/HeN)	20 Gy in 1fr. delivered by SARRP unit through two ventral beams	Morphological changes (H&E) Fibrosis (Masson Trichrome) Mast cells (toluidine blue staining)	Starting at 17 weeks after treatment	Pathological changes included fibrosis, inflammation, urothelial thinning, and necrosis. The radiation exposure attenuated the long-term urothelial integrity

*RT, radiotherapy; ICAM-1, intercellular adhesion molecule 1; mtNOS, mitochondrial nitric oxide synthase; COX, cyclooxygenase; UP-III, uroplakin-III; POMC, Proopiomelanocortin; HB-EGF, heparin-binding EGF-like growth factor; ICAM-1, irradiation on intercellular adhesion molecule 1; H&E, Hematoxylin & Eosin; SARRP, small animal radiation research platform*.

**Table 4:** Chronological summary of the pre-clinical studies about radioprotective effects on the normal bladder.

**Reference**	**Animal model (strain)**	**Set-up**	**Endpoint (method)**	**Toxicity timing after RT**	**Findings**
Edrees et al. (66)	Female mouse (C3H)	13–25 Gy in 1 fr. delivered by 250 kV X-ray machine + Cy	Micturition frequency (cystometry), incidence of haematuria	5 months (rad) 1 week (Cy) Early and 9–12 month (rad+Cy)	Cy administered up to 9 months before or after irradiation induced more severe bladder damage than X-rays alone
Malkinson et al. (67)	Male mouse (B_6_D_2_F_1_)	2–4.5 Gy/fr. x 10–15 fr. after PGs administration	Murine hair loss	Immediately after the fractionated RT	PGs may provide protection of tissue as bladder mucosa
Horsman et al. (68)	Female mouse (CDFl and C3H)	Nicotinamide injected after local irradiation delivered by 250 kV X-ray irradiator	i) Moist desquamation ii) Reservoir function (transurethral cystometry)	i) 11–30 daysii) 9 months	Best radiosensitization with minimal effect on normal tissues (bladder) at time of nicotinamide peak plasma drug concentrations
Kanai et al. (69)	Female rat (Sprague-Dawley)	35 Gy in 1 fr. delivered by 6 MeV linac + MnSOD transgene injection 24 h before RT	Transepithelial resistance and permeability damage on detrusor function	1, 48, and 96 h 7 and 24 days 6 months	MnSOD transgene allows transepithelial resistance and permeability to recover within 4 weeks and shows baseline pressures and more stable voiding patterns after 6 months
Jaal et al. (70)	Female mouse (C3H/Neu)	Graded radiation doses delivered by Seifert Isovolt 320/20 X-ray machine + rHuKGF	Reservoir function (transurethral cystometry)	Early phase response: 1–30 days Late phase response: 60–360 days	Early: ED50 from 20 to 27 Gy Late: ED50 from 16 to 22 Gy rHuKGF administration before irradiation modified early and late radiation effects
Dinçbaş et al. (71)	Male rat (Wistar)	25 Gy in 5 fr. delivered by Co^60^ teletherapy unit + AF + GEM	Bladder fibrosis (H&E)	4 months	AF may have a beneficial effect in limiting the radio-sensitizing effect of GEM
Rocha et al. (72)	Rat (sex n.a.) (Wistar)	11.64 Gy in 1 fr. delivered by 6 MeV linac + L-glutamine	Amount of collagen (Masson's trichrome, Picro Sirius Red) Immuno-histochemistry	15 days	L-glutamine seems to prevent bladder wall damage
Costa et al. (73)	Male rat (Wistar)	10 Gy in 1 fr. delivered by 10 MeV linac + L-arginine	Morphologic change of blood vessels in the wall (H&E, expression of VEGF and FGF)	16 days	L-arginine was radioprotective
Rajaganapathy et al. (51)	Female rat (Sprague-Dawley)	40 Gy in 1fr. delivered by SARRP unit + liposomal tacrolimus	Micturition frequency(cystometry) Morphological changes (H&E)	2 and 6 weeks	Lipo-tacrolimus treated rats show an increased post- irradiation IMI and minimal edematous changes
Horsman et al. (74)	Male and Female Mice (CDF1)	Graded radiation doses + VDA(CA4P)	Reservoir function (transurethral cystometry)	9 months	ED50 = 14 Gy for bladder VDA has no effect on the early (skin) or late (bladder and lung) tissues responding to radiation
Oscarsson et al. (75)	Female rat (Sprague-Dawley)	20 Gy in 1 fr. delivered by 6 MeV linac + with and without 20 sessions of HBOT	Oxidative stress and pro-fibrotic factors	28 days	HBOT may prevent radiation-induced changes
Sarsarshahi et al. (76)	Female mouse (C3H/Neu)	14-24 Gy in 1 fr. delivered by YXLON Maxishot device + bortezomib	Reservoir function (transurethral cystometry)	Acute response: 6–9 days Late response: 21–24 days	Daily bortezomib injections between days 0–15 resulted in a significant decrease in responders

*Several agents were tested in combination with radiation and the effect was measured using various techniques*.

*RT, radiotherapy; Cy, Cyclophosphamide; PGs, prostaglandins; MnSOD, Manganese superoxide dismutase gene therapy; VEGF, vascular endothelial growth factor; FGF, Wbroblast growth factors; AF, amifostine; GEM, gemcitabine; H&E, hematoxylin & eosin; rHuKGF, palifermin; HBOT, hyperbaric oxygen therapy; CA4P, combretastatin A-4 phosphate; VDA, vascular disrupting agents; SARRP, small animal radiation research platform; IMI, inter- micturition intervals IMI*.

**Table 5:** Chronological summary of the pre-clinical studies about bystander and abscopal effects: the clonogenic the survival of brain cells after pencil beam and/or microbeam *in-vivo* irradiation (usually using a synchrotron) is compared with that of the corresponding not-targeted bladder cells.

**Reference**	**Animal model (strain)**	**Set-up**	**Endpoint (method)**	**Euthanasia timing after RT**	**Findings**
Singh et al. (77)	Female mouse (C57BL6 and Balb/c)	Whole body irradiation (Co60 source) at single and serial low dose (20mGy-2Gy)	RIBE (clonogenic survival)	24 h	Genotype determined the type of bystander signal/response
Fernandez-Palomo et al. (78)	Rat (sex n.a.) (Wistar)	17.5, 35, 70, 350 Gy delivered by synchrotron on one brain hemisphere	RIBE (clonogenic survival)	4, 8, 12 h	Both MRT and HSR yielded a demonstrable abscopal effect after high doses of irradiation
Mothersill et al. (79)	Male rat (Wistar)	Whole body MRT and HSR on one brain hemisphere (35 and 350 Gy skin-entry doses)	RIBE (proteomics, clonogenic survival)	48 h (hours in cage with uneradicated rats)	Evidence of strong RIBE signal in the contra-lateral brain hemisphere and weaker effects in the distant bladder of the irradiated rats. Proximity to an irradiated animal induced signaling changes in an un-irradiated partner
Fernandez-Palomo et al. (80)	Male rat (Fisher)	MRT (20 or 200 Gy skin-entry doses) on one brain hemisphere with inoculated F98 cells	RIBE/abscopal effects (calcium flux, role of 5HT, clonogenic survival and proteomic profil)	48 h (hours in cage with unirradiated rats)	Membrane related functions were critical for true RIBE expression. Bystander effects (in partner animals) were not the same as abscopal effects (in the irradiated animal)
Fernandez-Palomo et al. (81)	Male/female mouse (NU-Foxn1^nu^)	PB (200 or 1,000 Gy skin-entry doses) and MRT (22 Gy or 110 Gy) on one brain hemisphere with and without glioma injected 7d earlier	RIBE/abscopal effects (calcium flux, clonogenic survival)	2, 12, 48 h	Calcium data did not support a calcium channel mediated mechanism. The presence of a tumor reduced or reversed the effect. The immune response played a role.

*Thus, in this field of research the normal bladder does not deal with any direct radiation effect*.

*RT, radiotherapy; RIBE, radiation-induced bystander effects; PB, Pencil Beam; MRT, microbeam irradiation; HSR, homogenous synchrotron radiation*.

### Contribution of Animal Models to the Understanding of the Physio-Pathogenesis of Radiation Cystitis

High energy ionizing radiation affects various bladder cell types, among which urothelial, neuronal, detrusor, and vascular smooth muscle cells; pre-clinical research in the last decades has tried to clarify these processes. At a molecular level, RT-induced injury can be triggered either via direct damage to DNA or other cellular macromolecules (i.e., protein, lipids etc.) causing early cell death and/or functional deficiency, or via an indirect activity, breaking down water atoms into free oxygen radicals and producing oxidative stress (82). The release of free oxygen radicals can cause cell membrane lipid peroxidation or react with DNA, leading in both cases to DNA damage, replication failure and cell death (3). Subsequently, a number of downstream abnormalities of the bladder wall might occur at multiple levels, which can be classified into three consequential phases: (a) an early or acute phase of inflammation, which occurs during or just after the completion of a conventional therapy protocol such as 2 Gy per 5 days/week to a total dose of 60–70 Gy in 6–7 weeks; (b) a symptom-free phase; (c) a late non-reversible, fibrotic phase that develops gradually and can be detected from 6 months to years after RT (48, 83). The former response is transient and often resolves in a few weeks or months. Its symptoms are caused by the activation of the pro-inflammatory cascade. In particular, one of the early stage events is the increase of the inflammatory, proliferative and pro-apoptotic nuclear factor-kappa B (NF-κB), which stimulates endothelial cyclooxygenase (COX2) expression and arachidonic acid conversion into prostaglandins in endothelial cells, determining vasodilatation and increased muscle tone (edema and hyperemia) (60). NF-κB activation might bring to the increase in membrane urothelial intercellular adhesion molecule 1 (ICAM-1) levels in the vascular endothelial cells, prompting and supporting leucocyte infiltration in the lesion (49, 50). These events result in a functional impairment of the organ, and patients therefore experience symptoms such as increased frequency, urgency and dysuria (47). After a symptom-free period, the duration of which is highly variable, a late chronic response might develop. In this phase, at molecular level, uroplakin 3 downregulation on the luminal surface of the bladder urothelium, together with loss of superficial urothelial cells (umbrella cells), produces the disruption of urine-plasma barrier and thus an increased permeability (59, 84, 85) leading to a chemical irritation of the bladder wall caused by urine components. Furthermore, transforming growth factor beta-1 (TGF-β1) expression increase, and the subsequent accumulation of extracellular matrix and collagen deposition eventually supports the development of fibrosis (86, 87).

Histologically, several phenomena can be detected, such as a combination of urothelial cell denudation and tumor-like epithelial hyperproliferation, vascular damage and hemorrhaging, submucosal telangiectasia, fibrin deposition, formation of ulcers, loss of smooth muscle cells, influx of fibroblasts, collagen accumulation and, eventually, fibrosis (17, 47). All these anomalies lead eventually to hematuria and a permanent reduction of the bladder compliance, which could ultimately result in an impaired ureteric emptying and, thus, renal dysfunction. Moreover, voiding failure can also derive from the progressive underactivity of detrusor muscle, which subsequently becomes acontractile.

Due to the complexity of this condition, current non-invasive treatment options have limited effectiveness and, in certain extreme scenarios, radical cystectomy is required (52).

The establishment of reliable preclinical models mimicking urothelial toxicity (UT) and aimed at understanding all the molecular processes involved in disease progression is fundamental for testing “tailored” therapies. To date, mice and rats have been commonly used for RC modeling, and a positive correlation has been seen between radiation dose (usually in the range of 5–40 Gy) and urothelial changes at early or late post-irradiation time points. However, different experimental methods, endpoints, irradiation doses, dose distribution and sources along with different animal species have been used. Therefore, the full comprehension of RT-induced UT and the availability of comprehensive models that faithfully recapitulate all the pathological paths still represent an unmet need.

Interestingly, dose and fractionation effects were mostly investigated in older studies (Table 2) using single fraction or minimally fractionated protocols. No studies on dose and fractionation using modern micro-irradiators have been published to date. Similarly, there are no specific studies dealing with the quantification of bladder volume effects and/or the existence of more sensitive sub-structures.

Each research group developed its own animal model using different strains of rats or mice, as listed in Tables 2–4. Despite this variability, the general practice was to use a single radiation dose of 20 Gy, roughly corresponding to a fractionated radiotherapy treatment delivering 60 Gy in 30 fractions over 6 weeks (4, 44). Several cystometric studies showed that 20 Gy is a dose sufficient to observe in at least 50% of animals (47, 49, 88), a biphasic response in the acute phase (88) at about 7 and 23 days after RT, respectively (47), and a late phase starting at 4–6 months (44, 49, 83). Interestingly, Dörr et al. highlighted a strong correlation between damage in the second (but not in the first) acute wave and late damage (47).

Some works also studied radiation doses over 20 Gy delivered in 1 fraction (41, 44, 47, 51, 89). This dose escalation was associated with a higher toxicity rate (41) and more severe symptoms in terms of bladder dysfunction (51), degenerative type of epithelial changes (41) and increase of mast cell density (44). Regarding the late response, the latent time was found to be inversely dependent on the dose (48, 89).

The effects of fractionated radiotherapy were investigated in several works (42, 48, 90). Dörr et al. demonstrated that when radiation is delivered in equal-sized dose fractions, the radiation dose producing the damage in 50% of animals (ED50) is higher than in single dose irradiation. Furthermore, ED50 was shown to be sensitive to the interval between fractions (90). Other studies highlighted that the sparing effect obtained by dose fractionation results in a lower risk of chronic response (42, 48).

Stewart et al. evaluated the recovery of bladder at late time points and the consequent re-irradiation tolerance in mice and highlighted a possible indirect correlation between long-term injury and radiation dose administered in the first treatment; furthermore the prolongation of the interval time between treatment did not prevent late radiation damage in the bladder (40).

### *In vivo* Functional Evaluation

As in clinical setting, also small animals functional assessment of radiation cystitis can be undertaken. Cystometric evaluation, in both mice and rats (Table 2) represents the state of the art for quantifying *in vivo* functional bladder impairment.

Historically, one of the first attempts at assessing urinary frequency was reported in 1978 by Stewart et al. by placing an irradiated mice in a metabolic cage and counting the number and size of urine patches on a paper moving under it (91). Subsequently more precise technologies have been developed (38). Various cystometric models exist, but catheterizing the animals and placing them in metabolic cages is generally necessary. The bladder catheter is connected to a pressure transducer and a microinjection pump. Micturition volumes are recorded with a fluid collector under the metabolic cage. A room-temperature saline solution can be instilled into the bladder constantly at different rates depending on the aims of the investigator. Thus, bladder basal pressure, threshold pressure, flow pressure, maximum micturition pressure, micturition volume and micturition interval can be directly recorded. When male mice or rats (which display slight differences among species) are used, due to the anatomic structure of the urethra in the rodents, surgical implantation of the catheter is generally necessary if bladder catheterization is required; the catheter is positioned at the dome of the bladder and then tunneled subcutaneously to an interscapular region incision (or, less frequently, to the abdomen).

Many examples of the application of this technology in radiation cystitis setting may be found in the literature (Table 2). Lundbeck et al. reported one of the first attempts in female mice (doses delivered from 5 to 40 Gy), showing a reduction of reservoir function starting from 20 Gy in both acute (peak at 14 days) and late phase (300 days follow up) (89). Stewart et al. ascertained an increased urinary frequency and a reduced bladder capacity in a mouse model (female mice of strain C3H) after irradiation from 10 to 30 Gy in both early and late time settings (6 to 53 weeks after treatment, each animal was examined every 4/6 weeks) (41). Vale et al. after irradiating four equal groups of nine female Wistar rats at 10, 15, 20 and 25 Gy, performed a weekly cystometric evaluation until 2 months after irradiation and subsequently once every 3 weeks up to 6 months. A biphasic reduction of at least 30% in the bladder compliance index (calculated as volume injected to induce an increase in intravesical pressure of 5 cmH20) was obtained at 4/6 weeks and at 6 months after irradiation in all groups of animals receiving at least 15 Gy (44). Dörr et al. evaluated bladder reservoir function in female mice through cystometry in the dose fractionation setting: four equal-sized doses per fraction with increasing intervals of 0–8 h were applied to female mouse (strain CH3) bladders, and bladder capacity was measured 3 times 2 weeks before irradiation and at 3–4 day intervals during the initial 30 days after irradiation, obtaining a clear dose-response relationship (44, 48, 90).

In conclusion, all the authors seemed to agree that acute damage is confined to only a few days after irradiation, irrespective of the dose delivered, while late toxicity could emerge at different time lapses and with intensity depending on radiation dose and fractionation; in addition, cystometry has to be considered as a feasible, easy to interpret and reliable way to assess RC functional impairment.

### Histopathological Model of RC

Several animal models, employing both mouse and rat, have been developed with the aim of investigating the pathological modifications that occur in the bladder after irradiation but a “standard” universally recognized RC model is still lacking. To standardize the evaluation of histologic patterns, which are meant to be surrogates of the functional status of the bladder, morphological scores have been used.

To date, hematoxylin and eosin (H&E), indisputably remains the most informative staining employed, allowing the recognition of macroscopic signs of both early acute and late histological changes. Rajaganapathy et al. described the alterations of the rats' bladder wall 6 weeks after radiation (early inflammatory phase) through an analysis of the organ sections stained with H&E. In their study it was possible to discriminate several pathological features at three different radiation doses (20, 30, and 40 Gy). No sign of inflammation could be detected at the lowest dose (20 Gy), while edematous changes, immune cell infiltration, ectasic blood vessels in the lamina propria and hyperplasic urothelium were evident following 30 Gy irradiation. In addition, the staining highlighted degenerative-type epithelial changes, urothelial cell swelling and small nests of urothelial cells in the lamina propria surrounding blood vessels after 40 Gy radiation (51). Zwaans et al. in their mouse model of chronic radiation-induced cystitis used a scoring method to show the presence of urothelial thinning, ischemic necrosis and inflammation on H&E-stained slide, while Masson trichrome staining, which allows for a better visualization of both collagen deposition and smooth muscle fibers, was employed to score fibrosis (52). Both an intensity-based score (52) or a percentage of bladder wall area score (92), have been used to assess fibrosis.

In order to support such histopathological evidences, immunohistochemical staining can be employed to better visualize features such as urothelium loss and loss of smooth muscle (e.g., with markers COX-1/2 and UP-III) (52, 65). Morphological scores have been implemented, for example, by Zwaans et al. using a simple “positive *vs*. negative” staining assessment (52), and by Jiang et al. using integrated optical density (92).

Many of the single histological features present in the animal models of RC, such as inflammatory infiltrate, submucosal fibrosis, surface ulceration and nests of urothelial cells within the lamina propria referred to as “pseudocarcinomatous urothelial hyperplasia,” have also been described in humans by several research groups (93, 94). Moreover, histopathological changes in the human irradiated bladder have also been divided into “early” (predominant <12 months after irradiation) and “late” (predominant >12 months after irradiation) changes, consistent with the proposed model of RC progression in the animal model (52, 93). However, there are some subtle differences: for example, fibrosis has seldom been reported in humans as an early change that persists into the chronic phase, while in small animal models it occurs in the late phase only (95). This implies that even given the extensive experimentation on animal models, there are some limitations in the application of this knowledge to humans to be considered when planning clinical trials and experimental treatments.

### Radioprotective Agents

Great effort has been spent in finding new radioprotective agents (RA) to improve the range of clinical options for the management of radiotherapy-induced toxicity. An RA is natural compound or an artificially synthetized substance able to prevent radiation induced acute and late effects. In other words, RA should protect patients' healthy tissues during treatment and prevent the development of detrimental effects (96). According to the timing of their administration, it is possible to distinguish three classes of RAs. The first class includes agents intended for the prophylaxis of RT injuries, and is therefore administered before exposure to the radiation dose (97). This category comprises compounds with sulfhydryl groups, antioxidant properties or free radical scavengers (98). The second class of RA is represented by mitigators, administered during or shortly after RT, before symptoms appear, and are aimed at minimizing toxicity by preventing or reducing radiation damage on cells or tissues (99). These mitigators are, in fact, directed at hindering a series of cellular insults that stimulate proliferation and immune-inflammatory responses, including DNA repair, apoptosis and regulation of signal transduction cascades (100). The third heterogeneous group comprises symptomatic treatments given after RT.

Currently, the clinical management of RC includes both systemic and local treatments mostly focused on pain and symptomatic relief, which, however, neither prevent the development of RC nor reverse it in case of assumption after RT administration. The approved therapies may vary depending on the degree and the phase of radiation induced bladder damage. Anticholinergic agents and β_3_-adrenergic receptor agonists, for instance, are used to attenuate acute phase symptoms such as frequency and urgency. On the other hand, a wide range of drugs are systemically administered to cope with RT induced chronic response. Examples of this class of pharmaceuticals are represented by WF10, also known as Tetrachlorodecaoxygen (TCDO), a formulation given intravenously able to stimulate natural immunity in order to reduce inflammation; sodium pentosan polysulphate (SPP), a synthetic sulphated polysaccharide that decrease urothelial permeability by replacing defective glycosaminoglycans; tranexamic acid, used to inhibit fibrinolysis and prevent clot urinary retention in patients with hemorragic cystitis (95).

Nevertheless, although systemic treatments are non-invasive and avoid inpatient hospital admission, these therapies had low efficacy often accompanied by dose-dependent toxicity. For this reason, local treatments and bladder irrigation are considered the first line of intervention in all grades of the disease (101), aiming at protect the urothelium, arrest focal bleeding points and remove blood clots. Several agents are employed as intravesical therapies and directed at improving bladder compliance (102) including formalin, aluminum salts, hyaluronic acid, prostaglandins, botulinum toxin, polydeoxyribonucleotides and early placental extract (83, 95).

In recent years, hyperbaric oxygen and laser ablation have also emerged as non-invasive management options able to produce symptom relief and stop the progression of the pathologic process. They are, however, cumbersome for patients, requiring lengthy treatments and, in case of ablation a performance status that typically patients with radiation-induced cystitis do not have (83, 95, 103).

To date, many compounds have promised improvements in preclinical radioprotection research, most belonging to the third treatment category of RAs. For instance, Bortezomib, a potent proteasome inhibitor currently used in clinics for multiple myeloma treatment, is implicated also in the blockade of NF-κB (104–108) and therefore it was recently investigated in a radiation induced urinary bladder dysfunction mouse model (76). The study employs a daily subcutaneous dose of 0.02 mg/ml of Bortezomib given between days 0–15 or 15–30, at the two acute radiation-induced bladder inflammatory waves, after a single graded radiation dose. The aim was to identify the window of time in which the drug was more effective. At cystometry evaluation the most favorable outcome was obtained in case of drug administration at the first acute inflammatory wave (days 0–15), with no significant variation when given in the second, meaning that distinct mechanisms are involved in the acute phases. In 2018, Ikeda et al. investigated the effect of the hormone relaxin in reversing radiation induced bladder fibrosis in adult female C57Bl/6 mice (109). Relaxin is a 6 kDa hormone involved in the relaxation of uterine smooth muscle and in the softening of the pubic symphysis during pregnancy. Although a relaxing effect of the hormone on the bladder has not been demonstrated yet, its receptors were found to be expressed on detrusor cells as well as in the lamina propria and, to a lesser extent, the urothelium. In this study, relaxin 2 was administered to 7-week post-irradiated animals (10 Gy radiation dose) at a concentration of 400 μg/kg/day for 2 weeks. As a result, the treatment increased bladder compliance and bladder wall force generation. The hypothesized mechanism of action involved the activation of specific pathways associated to the activation of relaxin receptors; the stimulation of neoangiogenesis through the phosphorylation of AKT, the expression of platelet derived growth factor (PDGF) and vascular endothelial growth factor (VEGF), the enhancement of contractile function mediated by increased Cav1.2 (i.e., L-type Ca^2+^ channel) and the arrest of profibrotic TGF-β signaling (110) induced by ERK1/2 phosphorylation and upregulation of neuronal Nitric Oxide Syntase (nNOS) and cyclic guanosine monophosphate (cGMP) levels. Tacrolimus, a calcineurin inhibitor that prevents the growth and differentiation of T cells by indirectly blocking IL-2 expression was tested in a RC rat model in which animals received a high radiation dose (40 Gy). In this study, due to its hydrophobic nature, in order to improve drug solubility and delivery and reduce systemic toxicity, Tacrolimus was encapsulated into liposome (51). A significant improvement on the inter-micturition interval was achieved (111). L-arginine, due to its anti-oxidant and anti-inflammatory properties (6, 112, 113), as well as due to its proposed protective effect on endothelium by the stimulation of endothelium-derived relaxing factors (114), was tested in several preclinical studies on pelvic radiation-induced bladder toxicity. In these studies, the amino acid administration triggered nitric oxide formation in animals with impaired endothelial function at basal levels (115), reduced radiation-induced diarrhea in around 40% of rats (116), and prevented bladder modification, restoring the morphology of blood vessels by recovering VEGF and FGF expression in the bladder wall (73).

## Conclusion and Future Perspectives

A brief summary of the revised literature is reported in Table 6, along with summarized conclusions. The current review has underlined renewed interest in pre-clinical research on radiation induced urinary toxicity and the bladder response to radiotherapy. In particular, a considerable interest in the development and testing of RA has been increased in recent years, especially for high risk conditions such the use of high doses, as for prostate cancer, and the existence of baseline risk factors, i.e., genetic predisposition or clinical factors (e.g., impaired baseline urinary function, adjuvant or salvage irradiation after prostatectomy). Importantly, a greater effort should be spent in the translation of pre-clinical results into clinical trials. Nonetheless, further pre-clinical studies are needed to clarify the applicability and therapeutic advantages of radioprotective agents in the treatment of radiation cystitis. Future goals will be the identification of novel molecules and strategies to pursue in order to guarantee a broader efficacy at a cellular, tissue, organ and whole organism level.

**Table 6:** Brief summary of the revised literature with some conclusions; unmet needs; future perspectives.

**Findings from literature**	**Conclusions**	**Future perspectives**
**Bladder mechanism**		
Radiation effect at the molecular level (direct and indirect damage to DNA) is followed by downstream **abnormalities of bladder** wall in three phases: 1) *Early/acute phase: reversible* → Increase of NF-κB → COX2 and prostaglandin expression → Vasodilatation, increased muscle tone → hyperemia, edema → increase of ICAM-1 → leucocyte infiltration → inflammatory symptoms (frequency, urgency, dysuria) 2) *Symptom-free phase* *Late phase: persistent, fibrotic* → UP-III downregulation and loss of umbrella cells → increase of permeability → chemical irritation from urine components → Increase of TGF-β1 expression → accumulation of extracellular matrix and collagen deposition → development of fibrosis → hematuria, permanent reduction of bladder compliance, voiding failure	The **full comprehension of RT-induced urothelial toxicity** and the availability of models that faithfully recapitulate all the pathological paths, both early and late phases, still represent an unmet need. The establishment of reliable preclinical **models mimicking urothelial toxicity** is fundamental for testing more “tailored” novel therapies.	Given the current interest in **hypo-fractionation and ablative therapies**, investigations mimicking extreme hypo-fractionation (e.g., radical doses delivered in 1–5 fractions) should help in better understanding the partially unknown mechanisms of bladder radiation response in these extreme situations. Experiments set to identify the mechanisms underlying **“spatial effects”** would be of paramount importance in possibly guiding plan optimization to selectively reduce the dose to these sub-structures.
**Animal models and dose set up:**		
•Each research group developed their own animal model using **different strains of rats or mice**. •Radiation dose was tested in the range **5–40 Gy**. •The general practice was to use a **single radiation dose of 20–25 Gy**, approximately equal to ED50 and estimated to mimic the delivered clinical doses to pelvic tumors. •**Doses over 20 Gy** proved to be associated with a higher toxicity rate and more severe symptoms. •ED50 increases with the **number of fractions** and the **interval between fractions**. •Late radiation injury seemed to be inversely related to the **dose given in the first treatment** and independent of the **interval between treatments**.	Very different experimental settings have been used: a “**standard**” universally recognized RC model is still missing. Even though a single dose of 40 Gy is well above the dose delivered in a clinical setting, the use of **high dose** can be useful for a better understanding of the underlying biological mechanisms.	Despite the availability of micro-irradiators with theoretically significant potentials for high-precision experiments, animal studies focused on gaining a better understanding of **dose, fractionation and volume effects** are largely lacking.**Reliable and reproducible methods** to quantify the **severity of RC** in animal models should be realized.
***In vivo*** **functional evaluation**		
•**Acute damage** is confined to only a few weeks after irradiation, irrespective of the dose delivered, with a biphasic response at about 7 and 23 days after RT, respectively. •**late toxicity** could emerge at different time lapses (within 6 month to 1 year) with intensity depending on radiation dose and fractionation. No changes in the **diurnal urinary pattern** were observed during cystometry. If **male mice/rats** are used, a surgical implantation of the catheter is deemed necessary.	Cystometry is the “state-of-the- art” objective tool in evaluating the ***in vivo*** response to radiation damage, in terms of **reservoir function** and/or **micturition frequency**.	**High-quality pre-clinical imaging platforms** are expected to extend the potential of **non-invasive** assessment of RC severity.
**Histopathological model of RC**		
•**H&E** (the most informative): recognition of both early acute and late histological changes. •**Masson trichrome**: to assess the level of bladder wall fibrosis as an intensity-based score or as a percentage of bladder wall area score. •**urothelial and inflammation markers** to better visualize the urothelium (e.g., COX-1/2 and UP-III). •Simple **“positive vs. negative” staining** using integrated optical density: to better visualize urothelium loss and loss of smooth muscle.	IHC is the gold standard for the **tracking** of disease progression in preclinical models. There are **limitations in the application of this knowledge to humans** that must be considered when planning clinical trials and experimental therapies.	The interaction between radiation induced reactions, damage repair and the immune system in the case of **combined immune-radiotherapy** is an extremely promising field of investigation, possibly involving several pelvic tumors.
**Radioprotective agents**		
*Clinical management of RC*: •**Systemic treatments** (e.g., anticholinergic agents and β3-adrenergic receptor agonists, TCDO, SPP, tranexamic acid): non-invasive and circumvent inpatient hospital admission; these therapies suffer from a very low efficacy, often accompanied by dose-dependent toxicity. •**Local treatments** and **bladder irrigation**: considered the first line of intervention in all grades of the disease, aiming at sterilization, arrest of focal bleeding points and removal of blood clots (e.g., intravesical therapies). •**hyperbaric oxygen** and **laser ablation**: emerged as non-invasive management; they are, however, cumbersome for patients requiring lengthy treatments, and a level of fitness that many patients with radiation cystitis do not possess.*Classes of RAs*: •agents for the **prophylaxis** of RT injuries, administered before exposure. •**mitigators** given during or shortly after RT, aimed at minimizing or preventing the effects of radiation on cells/tissues. •**treatments or therapeutic preparations** applied after RT, to ameliorate radio-induced symptoms.*Compounds in radioprotection preclinical research*: •**Bortezomib**: implicated in the NF-κB blockade. •**Hormone relaxin**: reversing fibrosis. •**Tacrolimus** and **L-arginine**: to hinder the production and release of pro-inflammatory cytokines.	RAs **improve the range of clinical options** for the management of the RT-induced toxicity in **combined therapies**.	We must expect the **translation of pre-clinical results into clinical trials** testing the protective effects of RAs, especially in situations where high doses need to be delivered (e.g., prostate cancer) and for patients at higher risk of toxicity due to genetic predisposition or clinical factors (e.g., the impaired baseline urinary function of patients irradiated after prostatectomy). Future goals will be the identification of **novel molecules and strategies** to pursue either alone or in combination in order to guarantee a broader efficacy at a cellular, tissue, organ and whole organism level.

*NF-κB, nuclear factor-kappa B; COX2, cyclooxygenase; ICAM-1, intercellular adhesion molecule 1; UP-III, uroplakin 3; TGF-β1, transforming growth factor beta-1; ED50, radiation dose producing the damage in 50% of animals; RC, radiation cystitis; reservoir function, reduction in the bladder capacity by >50% at a fixed intravesical pressure; H&E, hematoxylin and eosin; IHC, immunohistochemistry; RA, radioprotective agent; TCDO, Tetrachlorodecaoxygen; SPP, sodium pentosan polysulphate*.

Despite the availability of micro-irradiators, animal studies on bladder radiation focusing on dose, fractionation and volume effects, are largely lacking. Such investigations deserves greater attention for several reasons, e.g., given the growing interest in hypo-fractionation and ablative therapies, investigations mimicking these situations might help in better understanding the mechanisms of bladder radiation response in these extreme condition; or given some evidences (20) of high sensitivity to moderate hypofractionation (2.3–3.0 Gy/fr), experiments on animal models on this subject might shed light on the issue.

Another significant issue concerns evidence of “spatial” effects suggesting that specific sub-structures (i.e., the trigone) may be more sensitive to radiation (18–20): experiments set to identify the mechanisms underlying these effects would be of paramount importance in guiding plan optimization to selectively reduce the dose to these sub-structures.

A related development that could be useful for further advances in the field is the increasing use of combined therapies, including chemotherapeutic agents and immunotherapy. Testing dose and volume effects in these scenarios is an issue of paramount importance to understand the interaction between drugs and radiation induced reactions, damage repair and immune system.

Another largely unexplored field of investigation is pre-clinical imaging: high quality small animal images before and after radiation delivery may potentially become a powerful, non-invasive, quantitative surrogate for the measurement of radiobiological effects. For the bladder echography as well as targeted optical imaging seem especially promising.

Finally, an attempt should be made to set up reliable and reproducible methods to quantify the severity of RC in animal models. This implies the fulfillment of an easy-to-realize, ideally quick and economical, but at the same time sufficiently robust, quantitative analysis of RC severity both *in vivo*, e.g., by means of ultrasounds and/or MRI, and after the animal's sacrifice, e.g., through the development of a histological quantitative scoring of the radiation-induced lymphocyte infiltrate, collagen matrix deposition and neoangiogenesis.

## Author Contributions

SZ and ABre performed the literature review and wrote a large part of the manuscript and tables. AES contributed to the preclinical radiotherapy part and coordinated the work. GF, ABri, AS, and FM contributed to the anatomical description of the urinary tract. FS and RL contributed to the section on histopathology. ND, CF, and CC focused on the radiotherapy discussion. CF and CC also supervised the entire work. FB and RV contributed to the section focused on radioprotective agents. All authors contributed to the article and approved the submitted version.

## Conflict of Interest

The authors declare that the research was conducted in the absence of any commercial or financial relationships that could be construed as a potential conflict of interest.
